# Health Care Practitioners' Determinants of Telerehabilitation Acceptance

**DOI:** 10.5195/ijt.2020.6308

**Published:** 2020-06-30

**Authors:** Abdullah A. Almojaibel, Niki Munk, Lynda T. Goodfellow, Thomas F. Fisher, Kristine K. Miller, Amber R. Comer, Tamilyn Bakas, Michael D. Justiss

**Affiliations:** 1 Imam Abdulrhman Bin Faisal University (Iau), Saudi Arabia; 2 School of Health and Human Sciences, Indiana University, Indianapolis, IN, USA; 3 Lewis College of Nursing and Health Professions, Georgia State University, Atlanta, GA, USA; 4 Dwyer College of Health Sciences, Iu South Bend, Indiana, USA; 5 University of Cincinnati College of Nursing, Cincinnati, OH, USA; 6 School of Applied Health Sciences, Brooks Rehabilitation College of Healthcare Sciences Jacksonville University, Jacksonville, FL, USA

**Keywords:** Health care practitioners, Pulmonary rehabilitation, Respiratory care, Technology acceptance model, Telehealth, Telerehabilitation

## Abstract

**Background:**

Pulmonary rehabilitation is a multidisciplinary patient-tailored intervention that aims to improve the physical and psychological condition of people with chronic respiratory diseases. Providing pulmonary rehabilitation (PR) services to the growing population of patients is challenging due to shortages in health care practitioners and pulmonary rehabilitation programs. Telerehabilitation has the potential to address this shortage in practitioners and PR programs as well as improve patients' participation and adherence. This study's purpose was to identify and evaluate the influences of intention of health care practitioners to use telerehabilitation.

**Methods:**

Data were collected through a self-administered Internet-based survey.

**Results:**

Surveys were completed by 222 health care practitioners working in pulmonary rehabilitation with 79% having a positive intention to use telerehabilitation. Specifically, perceived usefulness was a significant individual predictor of positive intentions to use telerehabilitation.

**Conclusion:**

Perceived usefulness may be an important factor associated with health care providers' intent to use telerehabilitation for pulmonary rehabilitation.

Pulmonary rehabilitation (PR) is a multidisciplinary patient-tailored intervention that includes education, exercise training and behavior change, designed to improve the physical and psychological condition of people suffering from chronic respiratory disease (Spruit et al., 2013). PR can be beneficial to patients with chronic respiratory disease in a variety of ways including: reduction in dyspnea, improvement in exercise capacity, and improvement in mental health (Garvey et al., 2016; Ries et al., 2007). PR also has the potential to improve health-related quality of life, as well as reduce hospitalizations and decrease number of hospitalization days per patient (Ko et al., 2017).

It is challenging to provide PR services to the growing population of patients with chronic respiratory diseases due to shortages in health care practitioners and PR programs (Wade et al., 2014). Even in areas where PR programs are available, PR programs are underutilized (Liu et al., 2014). The reasons for low adherence rates have been reported to be poor access to a health care facility with a PR program, lack of transportation, and time constraints creating scheduling conflicts (Keating et al., 2011). New means of delivering health care services, such as telerehabilitation, have the potential to improve patients' participation and adherence with PR programs.

Telehealth is the use of telecommunication technology and electronic devices to enable remote clinical health care and health-related education (Doarn et al., 2014). While telerehabilitation is the use of telehealth to provide rehabilitation services such as physiotherapy, occupational therapy, speech therapy, and respiratory therapy to patients remotely (Tang et al., 2012). Multiple PR modalities, such as pursed-lip breathing technique training, supervised cardiopulmonary exercise, and disease-related education sessions could be provided via telecommunication technology for patients at home (Almojaibel, 2016).

Technology acceptance is a significant predictor of future use of telerehabilitation programs (Huis in 't Veld et al., 2010). Health care practitioners' acceptance of technology is the key factor affecting the success and sustainability of telerehabilitation programs (Wade et al., 2014). In fact, lack of rehabilitation staff acceptance of telerehabilitation has been cited as a significant potential barrier to implementation (Brewster et al., 2014). Understanding health care practitioners' acceptance of telerehabilitation may help to establish successful, higher quality, and safer telerehabilitation programs (Asaro et al., 2004). The factors influencing health care practitioners' intention to use telerehabilitation in PR programs are not well known. Specifically, there is a significant gap in the literature related to the factors influencing the intention to use telerehabilitation for PR among health care practitioners. This study used the technology acceptance model (TAM) to determine the influences of intention to use telerehabilitation among health care practitioners. This study had two hypotheses:

Perceived usefulness (PU) and perceived ease of use (PEOU) will have significant positive effects on the behavioral intention to use telerehabilitation.

Health care practitioner descriptors (age, working experience in PR, and PR program type) will have significant positive effects on the behavioral intention to use telerehabilitation.

## METHODS

A convenience sample of health care practitioners working in PR programs was recruited for participation from across the world. Sample size calculation was based on the number of responses needed to perform factor analysis, which recommends 5-10 times as many subjects as items in the scale (Ferketich, 1991). Based on the number of the TPRAS's items (17), the targeted number for enrollment for this study was between 85-170 participants. Participants were eligible if they: (1) read and write in English, and (2) are health care practitioners who were currently working in a rehabilitation center. Medical and health care professional students were excluded from participation in this survey. Data were collected by a self-administered Internet-based survey from January 2017 to May 2017 using the Tele Pulmonary Rehabilitation Acceptance Scale (TPRAS). TPRAS was developed previously from the TAM and showed strong evidence of content validity supported by nine experts (Almojaibel et al., 2019). The TPRAS consists of two subscales: Perceived usefulness (PU) and perceived ease of use (PEOU). In the context of using telerehabilitation, PU can be defined as the degree to which a person believes that using a telerehabilitation system would enhance his or her job performance. PEOU also can be defined as the degree to which a person believes that using a telerehabilitation system would be free of effort. Behavioral intention (BI) was this study's dependent variable. BI is the extent to which a potential user is ready to use a telerehabilitation system or the likelihood of using a telerehabilitation system.

REDCap (Research Electronic Data Capture) was used to provide information to interested participants about the study, collect participation consent, and collect data. REDCap is a free and secure web-based application designed to support the collection of anonymous responses for research studies (Harris et al., 2009, 2019). The electronic survey was provided to the potential participants via a hyperlink to the REDCap website. The survey's hyperlink was also sent to a group of health care practitioners society's email lists, Facebook pages, and via Twitter accounts, in addition to brochures sent to eight PR centers in the State of Indiana. After descriptor information was collected, participants were asked to either read a brochure or watch a video about telerehabilitation within the REDCap platform (see Appendix 1). Participants were able to access both means of information. The Indiana University Purdue University at Indianapolis Institutional Review Board (IRB) approved this study (protocol #1403903178).

**Figure 1.**
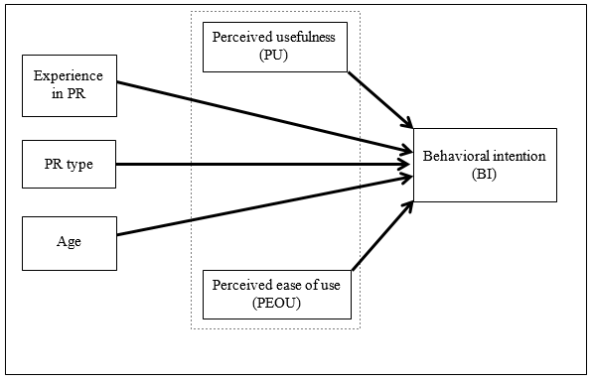
A model predicting health care practitioners' intention to use.

Multiple logistic regressions were conducted to examine the relationships between the predictors of telerehabilitation acceptance and the health care practitioners' intention to use telerehabilitation. The analysis included BI as the dependent variable while PU, PEOU, length of experience working in rehabilitation, program type, and age were included as the predictor variables. The dependent variable (BI), was dichotomized to Agree or Disagree based on the participants' responses on the 4-level Likert scale. First, the average score for each item of the BI subscale was calculated. Then scores above the midpoint values of 2.5 were categorized as positive intention, while scores equal or below 2.5 were categorized as negative intention. All the statistical analyses were conducted using the SPSS 24.0.0 software. Statistical significance for all of the analyses was set as p < .05. For the proposed relationships between the variables see Figure 1.

## RESULTS

A total of 222 health care practitioners working in PR programs completed the survey. The sample included health care practitioners from different health disciplines, including physicians, nurses, respiratory therapists, physiotherapists, occupational therapists, and exercise physiologists. The majority of the participants were respiratory therapists (55.7%). The responses were received from health care practitioners located in 29 different states in the United States of America and from another 20 countries across the world. Of the participants, 66.5% read the Telerehabilitation brochure and watched the Telerehabilitation examples video (Table 1). The majority of the participants (79%) indicated positive intention to use telerehabilitation. Item analysis showed that 36.9 % of the participants agreed that using telerehabilitation will improve patients' access to rehabilitation programs.

**Table 1.** Sample Characteristics of Health Care Practitioners in this Study

Characteristic	M	SD	Range
Age	40.44	12.09	21-68
Gender	**n**	%	
Female	120	54.1	
Male	83	37.4	
Preferred not to answer	19	8.6	
Location	**n**	%	
United States of America (U.S.A)	102 (29 States)	46	
Outside the U.S.A	46 (20 Countries)	20.7	
Not determined	74	33	
Type of the PR Program	**n**	%	
Hospital out-patient program	109	58.0	
Community-based program	15	8.0	
In-patient program	36	19.1	
More than one type of PR	28	14.9	
Health Care Profession	**n**	%	
Physician	15	7.4	
Nurse	17	8.4	
Respiratory therapist	113	55.7	
Physiotherapist	30	14.8	
Occupational therapist	5	2.5	
Exercise physiologist	18	8.9	
Other health care professional	5	2.5	
Experience in Rehabilitation Services	8.50	8.81	1-39 years

The reliability of the TPRAS was examined based on the participants' responses. The TPRAS's subscales (PU and PEOU) showed signs of internal consistency supported by a Cronbach's alpha of .92 and .80 respectively. The Cronbach's alpha of the BI subscale was .95.

Multiple logistic regressions were calculated on each of the significant variables identified through the regression analyses. Specifically, PU, PEOU, rehabilitation experience, program type, and clinician age were tested to determine their potential for a positive effect on BI to use telerehabilitation in the future. The analysis demonstrated a significant positive effect for only PU on clinician's BI to use telerehabilitation (? = 3.09, p < 0.01). All other considered variables were not predictive of clinicians' BI to use telerehabilitation (Table 2).

**Table 2.** Results of Regression Analysis to Examine Relationships Between PU, PEOU, Age, Work Experience in Rehabilitation, and Program Type and BI

Model	*B*	*p*	Odds Ratio	95% C.I. for *OR*	
				Lower	Upper
Perceived Usefulness (PU)	3.09	**< .01**	22.02	3.45	140.54
Perceived Ease of Use (PEOU)	1.27	.15	3.56	.623	20.39
Age	.02	.49	1.02	.957	1.10
Program Type (contrast variable: in-patient PR).		.57			
Hospital out-patient PR	−.81	.29	.45	.100	2.00
Community-based PR	19.13	1.00	202276279.90	.000	.
Experience in Rehabilitation	−.02	.61	.98	.891	1.07
Constant	−11.04	< .01	< .01		

Dependent Variable: BI.

## DISCUSSION

In our sample of health care practitioners, 79% indicated positive intention to use telerehabilitation in the future. The high percentage of health care practitioners willing to use telerehabilitation is a key finding for future telerehabilitation programs. The high percentage of telerehabilitation acceptance in our study does need to be interpreted with caution because 66% of the health care practitioners watched the telerehabilitation examples video and read the telerehabilitation brochure. The video and the brochure were used to demonstrate the concept of telerehabilitation in general. Liu and colleagues had similar findings with 68.24% of health care practitioners working in rehabilitation had positive intention toward using modern technologies (mechanical and computer systems) to improve patients' functions (Liu et al. 2015). Even though the telerehabilitation concept in Liu et al.'s study is different than the one introduced in our study, it is the only study that reported the percentage of telerehabilitation acceptance of health care practitioners before our study.

We examined multiple hypotheses about the relationships between the TAM constructs and the additional demographic variables. Similar to our findings, the PU was found to be a significant predictor of the positive intention to use telehealth or telerehabilitation in multiple studies (Hu et al., 1999; Kowitlawakul, 2011; Liu et al., 2015; Rho et al., 2014; Zailani et al., 2014). The effect of the PEOU on the intention to use telerehabilitation was not significant (B = 1.27, p < .15). The PEOU was found to be an insignificant predictor of telehealth or telerehabilitation acceptance in many other studies as well (Chen et al., 2015; Gagnon et al., 2012; Hu et al., 1999; Kowitlawakul, 2011; Zailani et al., 2014). Rho et al. (2014) found that the PEOU was a significant predictor of the positive intention to use telehealth among physicians. The participants in Rho et al.'s study were mainly from a capital city where the Internet is very common, and the participants were younger in age, which may explain why they have positive perceptions about telehealth ease of use. The variability on the PEOU significance can be explained by the difference between the proposed telehealth systems in each study and the difference between the populations. Previous studies that examined telehealth acceptance included participants from one or two health care professions. We measured telerehabilitation acceptance among different health care disciplines involved in PR. The participants in our study were expecting telerehabilitation to be difficult to learn. This suggests that, when introducing a new telerehabilitation program, health care organizations should first demonstrate the technology before the actual usage of the telerehabilitation. This feature study is unique in the literature because it reflects telerehabilitation acceptance of health professionals in modern pulmonary rehabilitation programs that are multidisciplinary.

## LIMITATIONS

There were several limitations in our study despite the important findings. The study sample size was relatively small considering the international reach and even though the survey was available online. However, the number of health care practitioners working in PR centers is very small. Most cardiopulmonary rehabilitation centers have one to four health care practitioners working in a cardiopulmonary rehabilitation center, and not all of them involved with the pulmonary rehabilitation program. A more targeted and personalized recruitment approach may have produced a more robust sample, as well as the provision of a monetary or similar incentive.

Another limitation of our study was the approach of introducing telerehabilitation to the participants. The only available method of introducing telerehabilitation was through showing the participants a short video demonstrating how it works with a copy of the telerehabilitation brochure that includes information about telehealth and its benefits. The participants had the choice to choose one method of review or the other. The way of introducing telerehabilitation to the participants may have provided an incomplete picture of the concept of telerehabilitation to the participants and may have affected their responses.

Also, using the online data collection method may have limited participation to those who were familiar with the telecommunication technology and using the Internet. This could have affected the percentage of the participants who were positive toward using telerehabilitation in the future because of their current usage of telecommunication technology.

## CONCLUSION

Using telerehabilitation is a relatively new method of delivering rehabilitation services. Telerehabilitation can be used to help PR programs improve access for patients living in rural areas and achieve outcomes by improving patients' adherence. Understanding the factors affecting potential users in their decision to use or not use telerehabilitation is a key factor to successful implementation of telerehabilitation programs. Therefore, we examined the factors of the positive intention to use telerehabilitation among health care practitioners.

Logistic regression analyses were conducted to identify significant telerehabilitation acceptance variables of health care practitioners working in PR. The results in this study confirmed that the TAM can be utilized in predicting telerehabilitation acceptance for health care practitioners. Perceived usefulness may be an important predictor of using telerehabilitation among healthcare practitioners. Potential telerehabilitation benefits (e.g., the ability to improve access to health care and improving patients' monitoring) were considered as the main perceived benefits of telerehabilitation. Additional variables such as age, experience in rehabilitation, and type of PR program increased the TAM predictability of positive intention to use telerehabilitation, but they were not significant factors.

This study measured the telerehabilitation acceptance of health care practitioners working in PR. The outcomes of our study explain telerehabilitation acceptance for key stakeholders planning to start telerehabilitation. Future studies might focus on modifying the TAM by adding new constructs that may improve the predictability of the model. Also, it will be beneficial if the sample included those who are not very familiar with the Internet by using face-to-face interview to answer the survey. Future studies could also consider adding a qualitative element so that the outcomes could more accurately capture practitioners' experiences and opinions regarding using telerehabilitation.
